# Staging intermediate hyperglycaemia for type 2 diabetes prevention: the ELSA-Brasil study

**DOI:** 10.1007/s00125-026-06743-0

**Published:** 2026-05-12

**Authors:** Paula A. Bracco, Maria I. Schmidt, Danilo de Paula, Jayne S. Feter, Michael Bergman, Bruce B. Duncan

**Affiliations:** 1 Postgraduate Program in Epidemiology, Universidade Federal do Rio Grande do Sul, Porto Alegre, Brazil; 2 Department of Statistics, Universidade Federal do Rio Grande do Sul, Porto Alegre, Brazil; 3 Department of Medicine, Holman Division of Endocrinology, Diabetes, Metabolism, NYU Grossman School of Medicine, VA New York Harbor Healthcare System, New York, NY USA; 4 Department of Population Health, Holman Division of Endocrinology, Diabetes, Metabolism, NYU Grossman School of Medicine, VA New York Harbor Healthcare System, New York, NY USA

**Keywords:** Clinical science and care, Epidemiology, Insulin sensitivity and resistance, Prediction and prevention of type 2 diabetes

## Abstract

**Aims/hypothesis:**

We aimed to evaluate stages in the development of type 2 diabetes by combining fasting plasma glucose (FPG) with 1 h plasma glucose (PG) or HbA_1c_ levels.

**Methods:**

Using data of 1174 middle- and older-aged Brazilian adults from the Rio Grande do Sul centre of the ELSA-Brasil cohort, 2017 to 2024, we developed stages based on previously recommended thresholds of mild (FPG: 5.6–6.0 mmol/l; 1 h PG: 6.7–8.5 mmol/l) and moderate (FPG: 6.1–6.9 mmol/l; 1 h PG: 8.6–11.5 mmol/l) hyperglycaemia. Similarly, we developed stages combining FPG levels with mild (39–41 mmol/mol; 5.7–5.9%) or moderate (42–46 mmol/mol; 6.0–6.4%) HbA_1c_ levels. We additionally assessed these staging schemas requiring an initial clinical score cutoff of ≥10% probability of developing diabetes in 10 years before proceeding to laboratory testing. We calculated baseline insulin responsiveness (insulin secretion-sensitivity index-2, ISSI-2) and the frequency of an estimated high risk of complications (Whitehall subphenotype clusters) across stages. We estimated relative risks of developing diabetes ascertained by self-report and glucose measurements after a 5.31 (0.44) year follow-up using robust Poisson regression with an offset for person-years. We calculated the prognostic properties of staging schemas in the prediction of diabetes.

**Results:**

The FPG/1 h PG schema stratified participants into three stages of decreasing ISSI-2 levels and increasing frequency of high risk for complications. The relative risk of incident diabetes increased progressively up to stage 3 in crude and adjusted models (15.4 [95% CI 6.1, 38.8] and 11.4 [4.5, 18.0]), respectively. This schema achieved 89.1% sensitivity and 53.7% specificity in the prediction of incident diabetes. When staging was applied with the clinical score, specificity improved (60.3%), and the need for laboratory testing decreased by 27.1%. The lower frequency of altered HbA_1c_ allowed for two-stage FPG/HbA_1c_ schemas, and produced lower relative risks and poorer prognostic properties compared with FPG/1 h PG schemas. Performance improved when combined with the clinical score. FPG/HbA_1c_ staging schemas were superior to the binary categorisation of prediabetes (intermediate hyperglycaemia).

**Conclusions/interpretation:**

FPG/1 h PG schemas resulted in a nuanced stratification of intermediate hyperglycaemia, with superior prognostic properties compared with FPG/HbA_1c_ schemas. Applying a predictive clinical score in staging reduced laboratory testing and false positives.

**Graphical Abstract:**

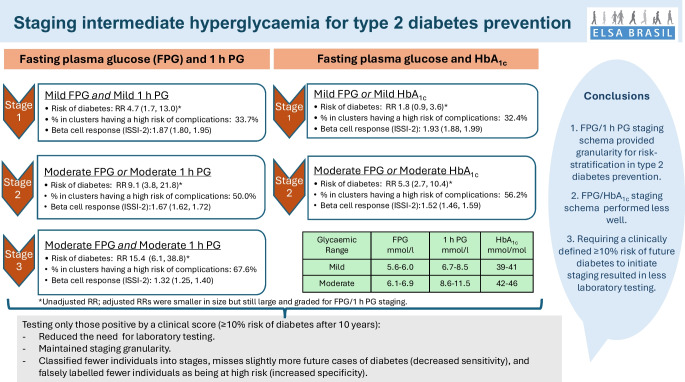

**Supplementary Information:**

The online version contains peer-reviewed but unedited supplementary material available at 10.1007/s00125-026-06743-0.


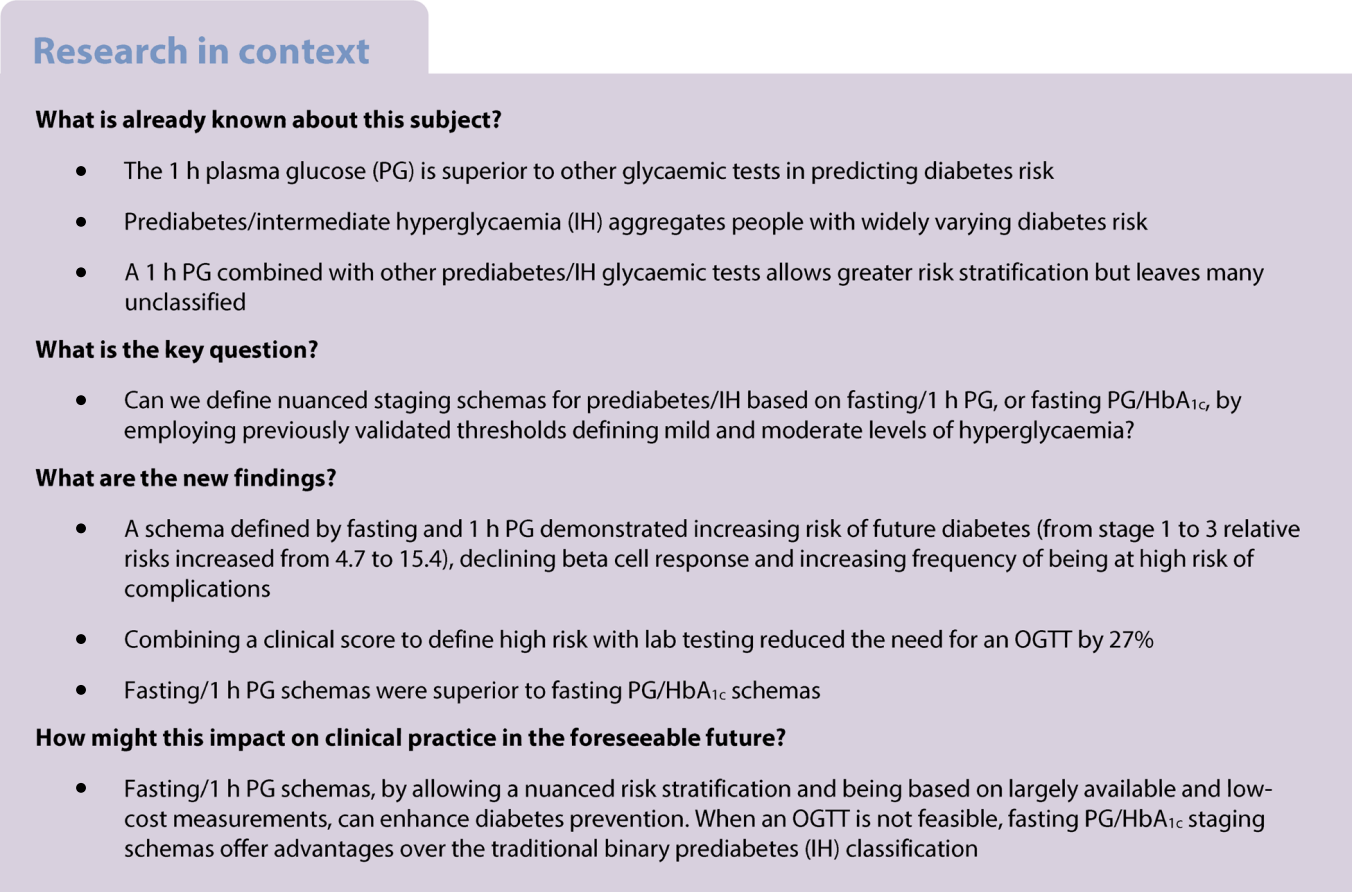


## Introduction

The onset of type 2 diabetes can be prevented by targeting and treating individuals at high risk with lifestyle interventions [1]. High-risk categories, widely referred to as prediabetes or intermediate hyperglycaemia (IH), account for approximately one billion adults globally [2], although their definitions are based on different tests and thresholds [3–5], translating into variable prevalences and rates of type 2 diabetes progression [6, 7] and related chronic complications [8]. With accumulating evidence that a 1 h plasma glucose (PG) ≥8.6 mmol/l (155 mg/dl) is more sensitive for predicting diabetes, risk of complications and mortality, the International Diabetes Federation (IDF) has recommended its use to characterise high risk in type 2 diabetes prevention [9].

Type 2 diabetes evolves over a long, heterogeneous and dynamic course of progressive alterations in both insulin sensitivity and beta cell function [10]. Despite this, high risk has been traditionally considered as a single prediabetes/IH category defined by fasting plasma glucose (FPG) or 2 h PG and HbA_1c_ thresholds. The excellent prognostic properties of the 1 h PG ≥8.6 mmol/l (155 mg/dl) stimulated its use in developing a two-stage schema for diabetes prevention, stage 1 being elevated 1 h PG and stage 2 its combination with traditional prediabetes/IH thresholds [11]. The schema demonstrated a progressive risk and a declining insulin responsiveness, but left unclassified 29.4% of people with prediabetes/IH as defined by the American Diabetes Association (ADA), as they did not meet the 1 h PG threshold [12].

Since it is unlikely that all four tests (FPG, 1 h and 2 h PG and HbA_1c_) will be used in staging prediabetes/IH in clinical practice, we considered dropping the 2 h PG, as the 2 h oral glucose tolerance test (OGTT) has been rarely performed in real-world settings [13]. We further reasoned that to develop more nuanced staging, we could use less stringent PG and HbA_1c_ thresholds in early stages and more stringent ones in later stages. To this end, to be practical, we chose well-known and previously validated thresholds for FPG [3, 4] and HbA_1c_ [5, 14] and two previously validated 1 h PG thresholds, ≥8.6 mmol/l (155 mg/dl) [9] and ≥6.7 mmol/l (120 mg/dl) [15].

Thus, using data from the Rio Grande do Sul centre of the ELSA-Brasil study, our objectives were: (1) to evaluate prognostic properties of FPG, 1 h PG and HbA_1c_ and their combinations in predicting future type 2 diabetes; (2) to develop and evaluate stages in the development of type 2 diabetes defined by FPG and 1 h PG levels, and, alternatively, by FPG and HbA_1c_ levels; and (3) to evaluate the utility of reserving laboratory testing for those with a clinical score indicative of a high risk of future diabetes.

## Methods

### Study design and sample

ELSA-Brasil is a prospective occupational cohort study assembled between 2008 and 2010 in six different Brazilian states. We enrolled active or retired civil servants of universities or research institutes aged 35–74 years. Participants completed a comprehensive set of questionnaires, clinical measurements and laboratory tests at the initial visit (Visit 1) [16, 17] and returned for three follow-up visits. The ELSA cohort is not representative of the Brazilian population since it consists of volunteers recruited from the employees of selected universities and research institutes. Participants all had stable jobs or pension plans and, in comparison with the general population, had greater educational attainment, more frequent white ethnicity and higher income.

**Fig. 1:**
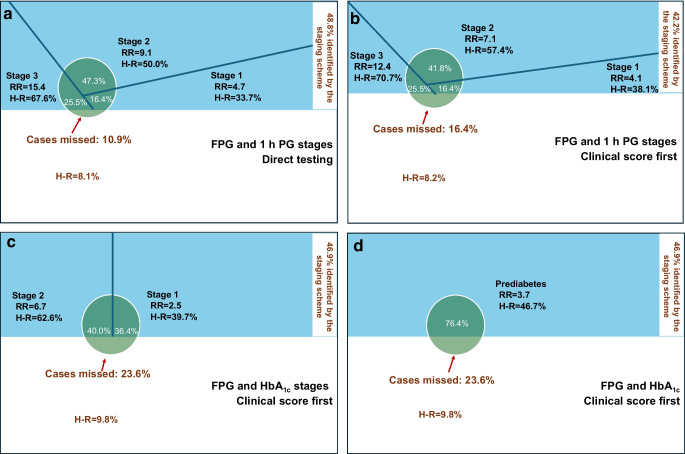
Diagrams comparing the prognostic properties of staging schemas. (**a**) Three-stage strategy based on combinations of mild and moderate elevations of FPG and 1 h PG. (**b**) Same strategy but initially requiring a ≥10% 10 year risk of developing type 2 diabetes determined by a clinical score (age, sex, self-declared ethnicity, BMI, waist circumference, hypertension and family history of diabetes) to initiate staging based on laboratory testing. (**c**) A two-stage strategy based on the presence of mild or moderate elevations of FPG and HbA_1c_. (**d**) A common approach to detect prediabetes with elevations of either FPG or HbA_1c_. Both FPG/HbA_1c_ strategies also required a positive clinical score to initiate staging. Mild and moderate hyperglycaemia were defined by previously validated thresholds (see text for threshold definitions). In each diagram, the blue areas represent the fraction of the sample in each stage, and the green circle represents those who developed incident type 2 diabetes over follow-up (5.7% of the sample). Numbers within this circle indicate the percentage of incident cases identified by each stage (stage sensitivity). RR, the relative risk of a participant developing type 2 diabetes over the 5.31 year follow-up; H-R, the percentage of participants in subphenotype clusters at high risk for diabetes complications. Area sizes are approximate

For the present analysis, we investigated participants at Visit 3 (2017–2019) at the Rio Grande do Sul centre (*n*=1741), the only visit and centre with 1 h PG included during a 2 h 75 g OGTT. As shown in electronic supplementary material (ESM) Fig. 1, we excluded people with a previous diagnosis of diabetes (*n*=288); with diabetes ascertained at Visit 3 (*n*=202) using standard WHO/ADA criteria for FPG, 2 h PG and HbA_1c_ as well as the IDF criterion of ≥11.6 mmol/l (209 mg/dl) for 1 h PG; and with missing glycaemic tests (*n*=77), thus leaving 1174 participants for baseline analyses. For longitudinal analysis, we also excluded individuals lacking information on race/ethnicity (*n*=7) and those with no follow-up (*n*=205), leaving 962 participants.

The study's research protocol received ethics committee approval, and all participants gave written consent to participate.

### Data collection and measurements

Certified technicians gathered information on age, sex, educational attainment, race/colour and use of medications, and measured weight, height, abdominal and hip circumferences, and blood pressure.

Following a 12 h fast, participants underwent a 75 g OGTT with fasting, 1 h and 2 h samples. We measured glucose and insulin locally using an enzymatic hexokinase method for glucose and an immunoenzymatic assay for insulin. Measurements were performed using Alinity reagent kits (Abbott, Lake Forest, IL, USA) according to the manufacturer’s instructions. The ELSA-Brasil central laboratory determined HbA_1c_ using a high-pressure chromatography method certified by the National Glycohaemoglobin Standardisation Program, and triglycerides and HDL-cholesterol using specific enzymatic methods [18].

### Variable definitions at Visit 3 baseline

We assessed beta cell compensation using the insulin secretion-sensitivity index-2 (ISSI-2), calculated as the product of (total AUC insulin/AUC glucose) × Matsuda Index, which, in our sample, satisfied the criteria for the hyperbolic relation of insulin secretion and sensitivity in individuals with normal glucose tolerance [19]. The Matsuda Index (MI) was based on fasting, 1 h and 2 h PG (mg/dl) and insulin (μU/ml) values using the formula MI = 10,000/√[(fasting glucose × fasting insulin) × (mean glucose × mean insulin)].

We characterised participants according to prediabetes subphenotype clusters indicative of high or low risk of cardiovascular and renal complications [20] using the calculator (https://cluster.apps.dzd-ev.org) developed for the Whitehall II cohort [21], based on age, sex, BMI, waist and hip circumferences, triglyceride/triacylglycerol, HDL-cholesterol, and glucose and insulin values during the 2 h OGTT.

Mild and moderate glycaemic elevations were defined as follows. For FPG, we considered moderate elevations to be the IH range recommended by the World Health Organization (WHO; 6.1–6.9 mmol/l;110–125 mg/dl) [3] and mild elevations to be the additional prediabetes range recommended by the ADA (5.6–6.0 mmol/l;100–109 mg/dl) [4]. For 1 h PG, we defined moderate elevations as the IDF prediabetes/IH range (8.6–11.5 mmol/l;155–208 mg/dl) [9] and mild elevations as the additional range beginning at the cutoff suggested by Abdul-Ghani et al (6.7–8.5 mmol/l;120–154 mg/dl) [15]. For HbA_1c_, we considered moderate elevations as the range recommended by the International Expert Committee (IEC; 42–46 mmol/mol; 6.0–6.4%) [5], and mild elevations as the additional prediabetes range recommended by the ADA (39–41 mmol/mol; 5.7–5.9%) [14].

### Diabetes outcome at Visit 4

We ascertained incident diabetes based on a physician diagnosis, use of diabetes medication(s) or a glycaemic measurement (FPG, 2 h PG or HbA_1c_) meeting standard WHO/ADA criteria for diabetes.

### Development of a type 2 diabetes staging schema for diabetes prevention

We evaluated Visit 3 thresholds for FPG, 1 h PG and HbA_1c_ and their combinations in the prediction of diabetes at Visit 4. To develop stages, we examined the risk of incident diabetes at Visit 4 in isolated and overlapping groupings of mild and moderate hyperglycaemia defined by FPG/1 h PG and FPG/HbA_1c_ at Visit 3 using Venn diagrams. For comparison, we also evaluated the risk for a dichotomous prediabetes/IH categorisation defined by an FPG ≥5.6 mmol/l (100 mg/dl) or an HbA_1c_ ≥39 mmol/mol (5.7%).

We additionally considered requiring a 10 year estimated probability of developing diabetes of 10% or greater before proceeding to laboratory testing. Each participant's probability was defined by a clinical score previously developed within the ELSA-Brasil cohort and based on age, sex, self-declared ethnicity, BMI, waist circumference, hypertension and family history of diabetes [22]. Only those meeting this clinical definition of high risk were staged based on glycaemic measurements; the remaining were classified as stage 0.

### Statistical analysis

Categorical variables were presented as absolute frequencies/percentages and continuous variables as arithmetic or geometric means, as appropriate. Baseline comparisons of ISSI-2 across stages were performed following multiple imputation for missing 1 h insulin values, with an *M* value of 30 and a maximum of 20 iterations.

We estimated relative risks (RRs) and their 95% CIs using robust Poisson regression with an offset for person-years. All models were performed with bootstrapping (1000 samples), with C statistics corrected for optimism and shrinkage factors applied to RRs to account for overfitting.

Correlation coefficients between glucose levels ranged from *r*=0.38 to 0.58, and between glucose and HbA_1c_, from *r*=0.21 to 0.26. Variance inflation factors for glycaemic markers were always less than 2.0, suggesting that multicollinearity was not present. In addition, to ensure that associations were not artefacts of categorisation, we modelled the risk of diabetes expressing glycaemic markers as continuous variables using restricted cubic splines. As seen in ESM Fig. 2, the predicted risk of diabetes increased across the spectrum of each glycaemic marker.

We evaluated the prognostic properties (sensitivity, specificity, positive predictive value and fraction labelled at risk) of glycaemic tests and their combinations, and for each stage and for each staging schema. A *p* value <0.05 defined statistical significance. We performed analyses with SAS version 9.4 (SAS Institute, Cary, NC, USA) and R software version 4.3.3 (R Foundation for Statistical Computing, Vienna, Austria).

## Results

### Sample description

Our baseline analytic sample (*n*=1174) had a mean (SD) age of 59.1 (9.1) years; 715 (60.9%) were women; most had a university degree; two-thirds were overweight or obese; and 681 (58.0%) had prediabetes/IH. The characteristics of those in our follow-up sample (*n*=962) were similar (ESM Table 1). Participants lacking glycaemic or insulin measurements were comparable to those having these measurements regarding age, BMI, waist circumference and FPG (ESM Table 2).

### Prognostic properties of glycaemic tests

After a mean (SD) follow-up of 5.31 (0.44) years, different tests and cutoffs, both isolated and in combination, yielded variable prognostic properties in predicting incident diabetes (Table 1). In isolation, the most sensitive (90.9% and 78.2%) tests were 1 h PG ≥6.7 mmol/l (120 mg/dl) and FPG ≥5.6 mmol/l (100 mg/dl), respectively, but their specificities were low (<50%). Requiring both criteria improved specificity to 61.1%; requiring higher levels of either FPG (≥6.1 mmol/l; 110 mg/dl) or 1 h PG (≥8.6 mmol/l; 155 mg/dl) improved specificity further (70.1%); and requiring both criteria at higher levels reached very high specificity (93.3%).

**Table 1:** Prognostic properties of different strategies to detect individuals at elevated risk of developing type 2 diabetes

Test	Based on incident diabetes (55 cases)				
	Sample labelled % (95% CI)	Sensitivity % (95% CI)	Specificity % (95% CI)	PPT+ % (95% CI)	PPT− % (95% CI)
Isolated					
1 h PG ≥6.7	60.9 (57.8–64.0)	90.9 (83.1–98.7)	40.9 (37.7–44.1)	8.5 (6.3–10.8)	1.3 (0.2–2.5)
1 h PG ≥8.6	28.3 (25.4–31.1)	63.6 (50.5–76.8)	73.9 (71.0–76.7)	12.9 (8.9–16.9)	2.9 (1.6–4.2)
FPG ≥5.6	51.8 (48.7–55.0)	78.2 (66.9–89.5)	49.7 (46.5–53.0)	9.7 (4.6–14.9)	5.1 (3.6–6.5)
FPG ≥6.1	11.8 (9.8–13.9)	34.5 (21.6–47.5)	89.5 (87.5–91.5)	16.7 (9.7–23.6)	4.2 (2.9–5.6)
HbA_1c_ ≥39	13.8 (11.6–16.0)	23.6 (12.0–35.2)	86.8 (84.6–89.0)	12.6 (8.1–17.0)	3.7 (2.4–5.1)
HbA_1c_ ≥42	4.8 (3.4–6.1)	7.3 (0.2–14.4)	95.4 (94.0–96.7)	8.7 (0.2–17.2)	5.6 (4.1–7.1)
2 h PG ≥7.8	8.7 (6.9–10.5)	30.9 (18.3–43.5)	92.6 (90.9–94.3)	20.2 (11.5–29.0)	4.3 (3.0–5.7)
1 h PG ≥6.7 and					
FPG ≥5.6	41.1 (37.9–44.2)	76.4 (64.8–88.0)	61.1 (57.9–64.3)	10.6 (7.6–13.7)	2.3 (1.1–3.5)
HbA_1c_ ≥39	10.4 (8.4–12.3)	23.6 (12.0–35.2)	90.4 (88.5–92.3)	13.0 (6.3–19.7)	4.8 (3.4–6.3)
1 h PG ≥6.7 or					
FPG ≥5.6	71.7 (68.9–74.6)	92.7 (85.6–99.8)	29.5 (26.6–32.5)	7.4 (5.4–9.3)	1.5 (0.1–2.9)
HbA_1c_ ≥39	64.3 (61.3–67.4)	90.9 (83.1–98.7)	37.3 (34.1–40.4)	8.1 (5.9–10.2)	1.5 (0.2–2.7)
1 h PG ≥8.6 or					
FPG ≥5.6	58.3 (55.2–61.4)	90.9 (83.1–98.7)	43.7 (40.4–46.9)	8.9 (6.5–11.3)	1.2 (0.2–2.3)
FPG ≥6.1	32.3 (29.4–35.3)	72.7 (60.6–84.9)	70.1 (67.1–73.1)	12.9 (9.1–16.6)	2.3 (1.1–3.5)
HbA_1c_ ≥39	36.6 (33.5–39.6)	74.5 (62.7–86.4)	65.7 (62.6–68.8)	11.6 (8.3–15.0)	2.3 (1.1–3.5)
HbA_1c_ ≥42	30.6 (27.6–33.5)	65.5 (52.5–78.4)	71.6 (68.6–74.5)	12.2 (8.5–16.0)	2.8 (1.6–4.1)
FPG ≥5.6 or					
HbA_1c_ ≥39	56.9 (53.7–60.0)	78.2 (66.9–89.5)	44.4 (41.2–47.7)	7.9 (5.6–10.1)	2.9 (1.3–4.5)
FPG ≥6.1 or					
HbA_1c_ ≥42	15.1 (12.8–17.3)	40.0 (26.6–53.4)	86.4 (84.2–88.7)	15.2 (9.3–21.1)	4.0 (2.7–5.4)
1 h PG ≥8.6 and					
FPG ≥5.6	21.8 (19.2–24.4)	50.9 (37.3–64.5)	79.9 (77.3–82.5)	13.3 (8.7–18.0)	3.6 (2.3–4.9)
FPG ≥6.1	7.8 (6.1–9.5)	25.5 (13.6–37.3)	93.3 (91.6–94.9)	18.7 (9.6–27.7)	4.6 (3.2–6.0)

ELSA-Brasil, Rio Grande do Sul (RS) centre, 2017–2019 to 2022–2024, *n*=962

FPG and 1 h PG are presented in mmol/l, and HbA_1c_ in mmol/mol

PPT+, probability post test+ (probability of developing diabetes over follow-up with a positive test result); PPT−, probability post test− (probability of developing diabetes over follow-up with a negative test result)

HbA_1c_ elevations in isolation were uncommon and thus had low sensitivity. Requiring either HbA_1c_ ≥39 mmol/mol (5.7%) or FPG ≥5.6 mmol/l (100 mg/dl) was more sensitive (78.2%) but had low specificity (44.4%); alternatively, requiring either HbA_1c_ ≥42 mmol/mol (6.0%) or FPG ≥6.1 mmol/l (110 mg/dl) had low sensitivity (40%) but high specificity (86.4%).

### Developing type 2 diabetes stages

Isolated and combined groups with mild and moderate ranges of glucose levels are shown in Venn diagrams, illustrating their size and RR of developing diabetes (ESM Figs 3, 4).

For FPG/1 h PG schemas, isolated mild hyperglycaemia (only one abnormal test; isolated portions of the top circles) compared with the reference group of normoglycaemia (FPG <5.6 mmol/l [100 mg/dl] and 1 h PG <6.7 mmol/l [120 mg/dl]) did not increase the risk of developing diabetes. When both tests indicated mild hyperglycaemia, the risk increased (RR 3.9). The risk was much higher when both tests indicated moderate hyperglycaemia (RR 12.7) (ESM Fig. 3).

In FPG/HbA_1c_ schemas, most participants with abnormalities had isolated mild or moderate FPG, with only 87 (9.0%) having mild and only 46 (4.8%) moderate levels of HbA_1c_. The RR of those having only mild elevations was considerably less than that of those with moderate elevations (ESM Fig. 4).

Based on these findings, for the FPG/1 h PG schema, we defined three mutually exclusive stages between normoglycaemia and diabetes: stage 1, requiring that both tests demonstrated mild hyperglycaemia; stage 2, where either test showed moderate hyperglycaemia; and stage 3, where both had moderate values. For the schema based on FPG and HbA_1c_, we identified two mutually exclusive stages between normoglycaemia and diabetes. The small overlap (36 participants; 3.7%) between mild FPG and mild HbA_1c_ precluded using this combination as a stage; thus, we defined stage 1 as having either FPG or HbA_1c_ at mild levels. For stage 2, we required reaching a moderate level of either FPG or HbA_1c_ (ESM Table 3). The minimal overlap (15 participants; 1.6%) having moderate levels of both FPG and HbA_1c_ precluded their characterisation as stage 3 (ESM Fig. 4).

### Evaluating staging schema based on FPG and 1 h PG

The ISSI-2 geometric means decreased approximately 50% across FPG/1 h PG stages with both direct testing and with a clinical score. The frequency of having a prediabetes subphenotype indicative of a high risk for complications rose 8.3-fold across stages with direct testing, and 8.6-fold when the clinical score was applied first, with more than two-thirds of the participants in stage 3 being classified as having a high risk of complications (Table 2).

**Table 2:** Geometric mean of beta cell function (ISSI-2) and frequency of being at high risk of complications according to staging schemas

Staging schema	*n*=1174	ISSI-2 Geometric mean (95% CI)	High risk of complications^a^ % (95% CI)
FPG/1 h PG schema			
Direct lab testing			
Stage 0 (reference)^b^	583	2.62 (2.56, 2.68)	8.10 (5.90, 10.3)
Stage 1	182	1.87 (1.80, 1.95)	33.7 (26.8, 40.6)
Stage 2	304	1.67 (1.62, 1.72)	50.0 (44.4, 55.6)
Stage 3	105	1.32 (1.25, 1.40)	67.6 (58.7, 76.6)
With a clinical score^c^			
Stage 0 (reference)	662	2.52 (2.46, 2.57)	8.20 (6.10, 10.3)
Stage 1	155	1.85 (1.77, 1.94)	38.1 (30.4, 45.7)
Stage 2	258	1.64 (1.58, 1.69)	57.4 (51.3, 63.4)
Stage 3	99	1.31 (1.24, 1.39)	70.7 (61.7, 79.7)
FPG/HbA_1c_ schema			
Direct lab testing			
Stage 0 (reference)	455	2.60 (2.53, 2.68)	11.0 (8.20, 13.9)
Stage 1	516	1.93 (1.88, 1.99)	32.4 (28.4, 36.5)
Stage 2	203	1.52 (1.46, 1.59)	56.2 (49.3, 63.0)
With a clinical score^c^			
Stage 0 (reference)	587	2.52 (2.46, 2.58)	9.80 (7.40, 12.2)
Stage 1	408	1.84 (1.79, 1.90)	39.7 (35.0, 44.5)
Stage 2	179	1.47 (1.40, 1.54)	62.6 (55.5, 69.7)
Prediabetes/IH^d^			
Direct lab testing			
Reference	455	2.60 (2.52, 2.68)	11.0 (8.20, 13.9)
Prediabetes/IH	719	1.81 (1.76, 1.85)	39.1 (35.6, 42.7)
With a clinical score^c^			
Reference	587	2.52 (2.45, 2.58)	9.80 (7.40, 12.2)
Prediabetes/IH	587	1.72 (1.67, 1.76)	46.7 (42.6, 50.7)

ELSA-Brasil, Rio Grande do Sul centre, *n*=1174

^a^Categorised by high-risk clusters identified by pathophysiology-based subphenotyping. For this specific outcome, six participants were excluded due to missing data

^b^Stage 0 includes 131 (13.6%) participants not meeting criteria for stage 1 but having prediabetes by ADA criteria

^c^Initially, with a clinical score requiring a ≥10% 10 year risk of type 2 diabetes to advance to laboratory testing; 261 (27.1%) did not reach this threshold

^d^As commonly defined by an FPG ≥5.6 mmol/l (100 mg/dl) or HbA_1c _≥39 mmol/mol (5.7%)

As shown in Table 3, the risk of future diabetes increased progressively across stages (for direct testing, from stage 1, RR 4.68; 95% CI 1.69, 13.0; to stage 3, RR 15.4; 95% CI 6.12, 38.8). When a clinical score was applied first, the RRs were slightly smaller; however, this approach avoided testing 261 (27.1%) individuals with low risk according to the clinical score. C statistics showed reasonable discrimination (~0.75) for models evaluating FPG/1 h PG schemas. When adjusting for covariates, the size of the associations decreased, but remained large, as did the gradients across stages. Plots showed good model calibration regarding the estimates of RR (ESM Fig. 5).

**Table 3:** Incidence, C statistic and RR of developing type 2 diabetes, according to staging schemas

Staging schema	Sample *n*=962	Diabetes									
		Cases *n*=55	Incidence %	Unadjusted				Adjusted^a^			
				C statistic		RR		C statistic		RR	
				Estimate	95% CI	RR	95% CI	Estimate	95% CI	RR	95% CI
FPG/1 h PG schema											
Direct lab testing				0.761	0.702, 0.819			0.809	0.780, 0.839		
Stage 0 (reference)^b^	493	6	1.2			1				1	
Stage 1	158	9	5.7			4.68	1.69, 12.96			3.91	1.47, 10.38
Stage 2	236	26	11.0			9.11	3.80, 21.84			7.43	3.08, 17.96
Stage 3	75	14	18.7			15.4	6.12, 38.78			11.41	4.50, 17.96
With a clinical score^c^				0.754	0.689, 0.819			0.793	0.760, 0.828		
Stage 0 (reference)	556	9	1.6			1				1	
Stage 1	135	9	6.7			4.11	1.66, 10.17			3.08	1.31, 7.23
Stage 2	201	23	11.4			7.11	3.35, 15.10			5.22	2.43, 11.19
Stage 3	70	14	20.0			12.37	5.57, 27.45			8.69	3.88, 19.45
FPG/HbA_1c_ schema											
Direct lab testing				0.669	0.593, 0.743			0.746	0.706, 0.789		
Stage 0 (reference)	415	12	2.9			1				1	
Stage 1	402	21	5.2			1.81	0.90, 3.63			1.45	0.74, 2.84
Stage 2	145	22	15.2			5.27	2.68, 10.35			3.40	1.74, 6.66
With a clinical score^c^				0.702	0.632, 0.777			0.754	0.714, 0.794		
Stage 0 (reference)	511	13	2.5			1				1	
Stage 1	321	20	6.2			2.46	1.24, 4.87			1.63	0.84, 3.18
Stage 2	130	22	16.9			6.68	3.47, 12.88			3.82	1.94, 7.54
Prediabetes/IH^d^											
Direct lab testing				0.614	0.556, 0.676			0.736	0.695, 0.779		
Reference	415	12	2.9			1				1	
Prediabetes/IH	547	43	7.9			2.73	1.46, 5.10			2.00	1.09, 3.69
With a clinical score^c^				0.657	0.599, 0.721			0.744	0.704, 0.786		
Reference	511	13	2.5			1				1	
Prediabetes/IH	451	42	9.3			3.67	2.00, 6.75			2.27	1.12, 4.15

ELSA-Brasil, Rio Grande do Sul centre, *n*=962

^a^Adjusted through robust Poisson regression for age (continuous), sex, education (three categories), race/colour (two categories: white/brown, black, Asian and indigenous), BMI (continuous) and a family history of diabetes

^b^Stage 0 includes 131 (13.6%) participants not meeting criteria for stage 1 but having prediabetes by ADA criteria

^c^Initially, with a clinical score requiring a ≥10% 10 year risk of type 2 diabetes to advance to laboratory testing; 261 (27.1%) did not reach this threshold, being placed in Stage 0

^d^As commonly defined by an FPG ≥5.6 mmol/l (100 mg/dl) or HbA_1c_ ≥39 mmol/mol (5.7%)

Table 4 shows prognostic properties of schemas in identifying future diabetes. When applied directly, the schema identified most cases (sensitivity 89.1%; 95% CI 80.6, 97.6), labelling 48.8% of the sample as high risk (stages 1–3). The more advanced stages 2 and 3 together identified 72.7%; (60.6, 84.9) of future cases in 32.3% of the sample. Initiating the schema with a clinical score resulted in slightly lower overall stage sensitivity (83.6%; 73.5, 93.7) while placing a lower fraction of the sample (42.2%) into stages 1–3.

**Table 4:** Prognostic properties of different staging schemas to detect individuals at elevated risk of developing type 2 diabetes

Stage	Fraction labelled % (95% CI)	Sensitivity % (95% CI)	Specificity % (95% CI)	Positive predictive value % (95% CI)
FPG/1 h PG schema				
Direct lab testing				
Stages 1, 2 and 3	48.8 (45.6–51.9)	89.1 (80.6–97.6)	53.7 (50.4–56.9)	10.4 (7.7–13.2)
Stages 2 + 3	32.3 (29.4–35.3)	72.7 (60.6–84.9)	70.1 (67.1–73.1)	12.9 (9.1–16.6)
Stage 3	7.8 (6.1–9.5)	25.5 (13.6–37.3)	93.3 (91.6–94.9)	18.7 (9.6–27.7)
Stage 2	24.5 (21.8–27.3)	47.3 (33.7–60.9)	76.8 (74.1–79.6)	11.0 (7.0–15.0)
Stage 1	16.4 (14.1–18.8)	16.4 (6.3–26.5)	83.6 (81.2–86.0)	5.7 (2.0–9.3)
With a clinical score^a^				
Stages 1, 2 and 3	42.2 (39.1–45.3)	83.6 (73.5–93.7)	60.3 (57.1–63.5)	11.3 (8.2–14.4)
Stages 2 + 3	28.2 (25.3–31.0)	67.3 (54.5–80.1)	74.2 (71.3–77.1)	13.7 (9.5–17.8)
Stage 3	7.3 (5.6–8.9)	25.5 (13.6–37.3)	93.8 (92.3–95.4)	20.0 (10.4–29.6)
Stage 2	20.9 (18.3–23.5)	41.8 (28.4–55.3)	80.4 (77.8–83.0)	11.4 (7.0–15.9)
Stage 1	14.0 (11.8–16.2)	16.4 (6.3–26.5)	86.1 (83.9–88.4)	6.7 (2.4–10.9)
FPG/HbA_1c_ schema				
Direct lab testing				
Stages 1 and 2	56.9 (53.7–60.0)	78.2 (66.9–89.5)	44.4 (41.2–47.7)	7.9 (5.6–10.1)
Stage 2	15.1 (12.8–17.3)	40.0 (26.6–53.4)	86.4 (84.2–88.7)	15.2 (9.3–21.1)
Stage 1	41.8 (38.7–44.9)	38.2 (24.9–51.4)	58.0 (54.8–61.2)	5.2 (3.1–7.4)
With a clinical score^a^				
Stages 1 and 2	46.9 (43.7–50.0)	76.4 (64.8–88.0)	54.9 (51.7–58.2)	9.3 (6.6–12.0)
Stage 2	13.5 (11.3–15.7)	40.0 (26.6–53.4)	88.1 (86.0–90.2)	16.9 (10.4–23.5)
Stage 1	33.4 (30.4–36.4)	36.4 (23.2–49.5)	66.8 (63.7–69.9)	6.2 (3.6–8.9)
Prediabetes/IH^b^				
Direct lab testing				
Single stage	56.9 (53.7–60.0)	78.2 (66.9–89.5)	44.4 (41.2–47.7)	7.9 (5.6–10.1)
With a clinical score^a^				
Single stage	46.9 (43.7–50.0)	76.4 (64.8–88.0)	54.9 (51.7–58.2)	9.3 (6.6–12.0)

ELSA-Brasil, Porto Alegre centre, 2017–2019 to 2022–2024, *n*=962 (55 incident cases)

Positive predictive value: probability of developing type 2 diabetes over follow-up

^a^Initially, with a clinical score requiring a ≥10% 10 year risk of type 2 diabetes to advance to laboratory testing

^b^As commonly defined by an FPG ≥5.6 mmol/l (100 mg/dl) or HbA_1c_ ≥39 mmol/mol (5.7%)

### Evaluating staging schema based on FPG and HbA_1c_

The ISSI-2 values declined by approximately 40% from stage 0 to 2 for FPG/HbA_1c_ approaches both with and without the clinical score (Table 2). In the single prediabetes/IH category, ISSI-2 values were approximately 30% lower than those in the reference group. The gradient across stages of having a high risk of complications was also smaller (approximately five- to sixfold) than seen in FPG/1 h PG schemas and even smaller (approximately fourfold) considering just the single category of prediabetes/IH.

As seen in Table 3, the risk of future diabetes increased minimally at stage 1 (RR 1.81; 95% CI 0.90, 3.63) and up to RR 5.27 (2.68, 10.35) at stage 2. When the staging schema was applied after a positive clinical score, the RRs for diabetes across stages improved (e.g. stage 2 RR 6.68; 3.47, 12.9), but were still lower than those in FPG/1 h PG schemas. Considering prediabetes/IH as a single category, the adjusted RR when including the clinical score was 3.67 (2.00, 6.75). C statistics indicated worse performance of FPG/HbA_1c_ staging models than models for the FPG/1 h PG schemas. The size of RRs also decreased considerably after adjustment for covariates (Table 3).

As seen in Table 4, the prognostic properties of this FPG/HbA_1c_ schema were inferior to those of FPG/1 h PG schemas in terms of sensitivity, specificity and sample labelling. Stage 1 was remarkably uninformative: only 5.2% (95% CI 3.1, 7.4) of its participants had a high risk of future diabetes (positive predictive value). Initiating the scheme with a clinical score improved specificity, thereby reducing the number of individuals labelled in staging. However, those in stage 1 still had a very low (6.2%; 3.6, 8.9) risk of progressing to diabetes. The single prediabetes category, when applied in conjunction with a clinical score, had a sensitivity of 76.4% (64.8, 88.0) and a specificity of 54.9% (51.7, 58.2), labelling 46.9% of individuals as high risk.

### Individuals not meeting criteria for the FPG/1 h PG schema

With the FPG/1 h PG staging schema, 92 people who had prediabetes/IH by FPG (ESM Fig. 3) did not meet the 1 h PG cutoff for stage 1. An additional 39 individuals with prediabetes by other ADA criteria (2 h PG or HbA_1c_) also did not meet stage 1 criteria. Summed together, these 131 (13.6%) individuals with prediabetes by ADA criteria were ‘unclassified’ by the FPG/1 h PG staging schema. Their RR of future diabetes, compared with those with normoglycaemia, was 0.55 (95% CI 0.07, 4.68), justifying their classification in stage 0.

In addition, 130 participants with 1 h PG values of 6.7–8.5 mmol/l (120–154 mg/dl) but FPG <5.6 mmol/l (100 mg/dl) also did not meet the criteria for stages 1–3. Their RR of progressing to diabetes was 0.52 (95% CI 0.06, 4.63), also justifying their classification in stage 0. Of note, only six participants in stage 0 developed diabetes during follow-up; none had prediabetes by other criteria (impaired glucose tolerance or HbA_1c_ ≥39 mmol/mol).

### Overall evaluation of staging schemas

The main points of the schemas evaluated are summarised in Fig. 1.

First, FPG/1 h PG strategies, direct laboratory testing (Fig. 1a) or initiating with a clinical score (Fig. 1b), were very sensitive, missing fewer future cases of diabetes (10.9% and 16.4%) than the FPG/HbA_1c_ staging (Fig. 1c) or the traditional single prediabetes category (Fig. 1d). Second, the more advanced FPG/1 h PG stages identified individuals at very high risk of progression to diabetes (e.g. with direct testing, RRs 15.4 and 9.1), much higher than found with FPG/HbA_1c_ approaches. Similarly, more than two-thirds of individuals in the most advanced FPG/1 h PG stage had a high estimated risk of developing complications, a considerably greater fraction than 46.7% found for the standard single prediabetes/IH category. Third, when stratified into stages by the FPG/1 h PG schema, participants presented a more nuanced gradient of risk (e.g. with direct testing, RRs ranging from 4.7 to 9.1 to 15.4) than with the FPG/HbA_1c_ approaches. Fourth, these benefits were achieved despite FPG/1 h PG staging labelling slightly fewer samples as at high risk, as can be seen when comparing Fig. 1b and c.

## Discussion

In this population of Brazilian adults, the FPG/1 h PG staging schema, which combines two traditional threshold values for FPG with two cutoffs for 1 h PG, distinguished progressive stages between normoglycaemia and diabetes. These stages demonstrated declining beta cell compensation, increasing percentage of individuals with high risk of complications and graded risk of future diabetes. In addition, staging had high overall sensitivity in detecting incident diabetes, with a reasonable (<50%) fraction of the sample being classified into stages of progressive risk. Its application with a clinical score reduced the need for laboratory testing by 27%. FPG/1 h PG schemas had superior properties compared with FPG/HbA_1c_ schemas.

A previously proposed staging schema using a 1 h PG ≥8.6 mmol/l (155 mg/dl) in isolation (stage 1) and in combination with elevated FPG, 2 h PG or HbA_1c_ levels (stage 2) [11] demonstrated progressive deterioration across stages, with declining insulin sensitivity and beta cell compensation and a substantially increased risk of future diabetes. However, 29.4% of individuals presenting prediabetes/IH by conventional testing were not classified [12].

By adding an initial stage based on a 1 h PG ≥6.7 mmol/l (120 mg/dl) threshold in our FPG/1 h PG schema, we classified as stage 1 many people with prediabetes/IH who would have been unclassified if staging was initiated with a 1 h PG value of 8.6 mmol/l (155 mg/dl) [12, 23]. Considerably fewer (13.6%) individuals with prediabetes/IH by ADA criteria were unclassified, most having an isolated elevated FPG and low risk of future diabetes (ESM Fig. 3). Furthermore, by combining two ranges of 1 h PG with two ranges of FPG defined by widely known and validated thresholds, we gained granularity in staging.

We found no studies evaluating stages based on HbA_1c_ and FPG levels. Our findings indicated that staging based on FPG/HbA_1c_, although inferior to FPG/1 h PG staging, represents an advance over a single category of prediabetes/IH, with the risk of diabetes in stage 2 almost doubling that of the single prediabetes/IH category. The greater specificity that resulted from applying FPG/HbA_1c_ staging after a clinical score decreases the need for laboratory testing and improves risk prediction. However, given the much larger fraction of the sample with abnormal FPG than with abnormal HbA_1c_, flexibility in creating stages was limited, with stage 1 accommodating >70% of individuals staged but portending little risk of future diabetes.

While the FPG/HbA_1c_ schema has the advantage of being based on a single blood sample without the need for an OGTT, our findings demonstrate that the FPG/1 h PG staging schema provides more accurate and nuanced predictions of future diabetes as well as stronger gradients of decreasing beta cell compensation and risk of chronic complications. Of note, the superiority of the 1 h PG vs FPG/HbA_1c_ for the diagnosis of type 2 diabetes was recently documented in a meta-analysis [24].

Within a pathophysiological framework, the combination of 1 h PG and FPG captures a broad set of biological mechanisms driving the development of type 2 diabetes. Specifically, FPG captures the effect of hepatic insulin resistance, and 1 h PG captures effects of impaired early and late phase insulin secretion and that of an accelerated rate of glucose absorption, among others leading to hyperglycaemia and diabetes [25, 26].

### Study limitations and strengths

Some study limitations merit consideration. Our relatively small sample may generate unstable estimates, and, thus, we used bootstrapping to strengthen internal validity. Although differences in Whitehall II and ELSA-Brasil participants raise an issue of calibration regarding our estimates of the frequency of a high risk of complications, calibration is less important in assessing gradients in this frequency across stages. Our ascertainment of diabetes at follow-up did not include the 1 h PG, which probably resulted in an underestimation of risk and of prognostic properties for FPG/1 h PG stages. In addition, the probability of developing diabetes in each stage (post-test probability) depends on its incidence, which varies across countries and population characteristics. Thus, to enhance generalisability, we compared the clinical significance of stages not with the absolute probability of developing diabetes but with the corresponding, more generalisable, RR. However, external validation of the staging schema in other populations and settings is needed. Finally, that our sample is an occupational cohort of predominantly white civil servants with stable employment and high educational attainment may limit the generalisability of our findings.

The strength of our study lies in its foundation on a contemporary cohort comprising both men and women with a broad range of ages, educational attainment and adiposity.

### Implications

Our study has significant clinical and public health implications. Globally, over a billion adults are estimated to have prediabetes/IH [2]. Additionally, 20–40% of young adults presently free of diabetes will probably develop the disease during their lives if further action is not taken [27, 28]. To curb this rising epidemic of type 2 diabetes, both population and clinical strategies are needed [29].

Clinical strategies targeting high-risk individuals, although proven effective, are infrequently implemented in formal prevention programmes, probably in part due to the limited benefits shown to-date in community samples. For example, a meta-analysis of lifestyle interventions in community trials found an absolute benefit for only three out of every 100 participants receiving the intervention [1]. This limited benefit is in part due to the lesser reduction in risk produced by the lesser intensity of lifestyle interventions of community trials. However, it is also largely due to many who receive these interventions being at low risk, given the inability of prediabetes to identify those who are most likely to progress to diabetes and thus to benefit most from lifestyle interventions. Furthermore, with the increasing availability of more effective nutritional and pharmacological approaches, identifying those at the highest risk becomes increasingly relevant. FPG/1 h PG staging provides the granularity needed to enable an earlier, more accurate and more personalised identification of individuals at risk for a stepped approach in type 2 diabetes prevention.

Given the recency of the IDF recommendation [9], the 1 h test is still infrequently performed in diabetes prevention. Although the 1 h test entails less effort than the 2 h OGTT, it does involve more effort than a single HbA_1c_, and its implementation will therefore face resistance. However, with its low cost and the increasing evidence of its benefits, greater use of the 1 h test could represent a major advance in diabetes prevention, analogous to that obtained with the near universal application of the OGTT in preventing complications of gestational diabetes.

The role of HbA_1c_ testing in type 2 diabetes prevention is open to debate, particularly considering its lesser pathophysiological basis. Additionally, HbA_1c_ is influenced by ethnic variability [30], anaemia and haematological conditions [31]. Furthermore, its costs and the requirement for a more challenging standardisation [32] compared with PG have made it less accessible in low- and middle-income settings, where approximately 95% of the projected increase in diabetes cases is estimated to occur by 2050 [2]. Similarly, while more extensive and sophisticated phenotyping for stage classification can be developed [20] and methods to detect high risk based on continuous glucose monitoring may soon become available, many low- and middle-income countries will have limited ability to adopt these options, constrained by their cost.

In conclusion, although both schemas, FPG/1 h PG and FPG/HbA_1c_, have their pros and cons, FPG/1 h PG staging yielded a more sensitive and specific characterisation of the progressive preclinical course of type 2 diabetes. Staging schemas that first assess a clinical score and then conduct laboratory testing reduced the need for testing and classified fewer individuals erroneously as being at high risk of diabetes.

## Supplementary Information

Below is the link to the electronic supplementary material.

ESM (PDF 716 KB)

## Data Availability

The data are not publicly available due to privacy or ethical restrictions. The data that support the findings of this study are available from the corresponding author on reasonable request, subject to necessary ethical approval and agreement by the ELSA-Brasil Steering Committee, represented by Maria Inês Schmidt and Bruce B. Duncan. Data are typically shared for the duration of the approved analysis through secure transfer mechanisms.

## Abbreviations

ADA: American Diabetes Association
FPG: Fasting plasma glucose
IDF: International Diabetes Federation
IH: Intermediate hyperglycaemia
ISSI-2: Insulin secretion-sensitivity index-2
MI: Matsuda Index
OGTT: Oral glucose tolerance test
PG: Plasma glucose
RR: Relative risk
WHO: World Health Organization
