# Supplementary material for: Staging intermediate hyperglycaemia for type 2 diabetes prevention: the ELSA-Brasil study
Source: Diabetologia. 2026 May 12;69(8):2212–24. doi: 10.1007/s00125-026-06743-0 (PMC13310221; doi:10.1007/s00125-026-06743-0)
Supplement: Supplementary file 1 — ESM (PDF 716 KB) [file 125_2026_6743_MOESM1_ESM.pdf]

Electronic Supplemental Materials (ESM)

This document provides supplementary material for the manuscript: Bracco P et al. Staging intermediate hyperglycaemia for type 2 diabetes prevention: the ELSA-Brasil study.

Table of Contents

ESM Table 1 – Sociodemographic and clinical characteristics of the study sample. ELSA-Brasil (Rio Grande do Sul center), visit 3 (2017-2019). 2

ESM Table 2 – Comparison of those excluded due to missing laboratory with those not excluded. 3

ESM Table 3 – Stages of type 2 diabetes based on fasting plasma glucose (FPG) and 1 h plasma glucose (1 h PG) or FPG and glycated haemoglobin (HbA_{1c}) 4

ESM Fig. 1 – Study sample flow diagram. 5

ESM Fig. 2 – Risk of incident diabetes across the spectrum of each glycemic marker used to create stages. A) fasting plasma glucose; B) 1 h plasma glucose; C) HbA_{1c}. 6

ESM Fig. 3 7

ESM Fig. 4 8

ESM Fig. 5 – Calibration plots for models of stages in the prediction of incident diabetes for the four approaches to staging. 9

ESM Table 1 – Sociodemographic and clinical characteristics of the study sample. ELSA-Brasil (Rio Grande do Sul center), visit 3 (2017-2019).

Characteristic	Baseline N = 1174 N (%) or mean (SD)	Follow-up N = 962 N (%) or mean (SD)
**Female sex, n (%)**	715 (60.9%)	606 (63.0%)
**Age, mean (SD)**	59.1 (9.1)	58.3 (8.9)
**White skin colour self-reported, n (%)**	911 (78.1%)	759 (78.9%)
**Educational attainment, n (%)**		
Less than secondary	124 (10.6%)	84 (8.7%)
Complete secondary	271 (23.1%)	219 (22.8%)
Complete university	779 (66.4%)	659 (68.5%)
**Body mass index, n (%)**		
< 25.0 kg/m^2	430 (36.6%)	355 (36.9%)
25.0 to < 30.0 kg/m^2	477 (40.6%)	394 (41.0%)
≥ 30.0 kg/m^2	267 (22.7%)	213 (22.1%)
**Abdominal obesity,† n (%)**	590 (50.3%)	484 (50.3%)
**Prediabetes,‡ n (%)**	681 (58.0%)	574 (56.9%)
**Glycaemic metrics, mean (SD)**		
Fasting plasma glucose, mmol/L	5.56 (0.46)	5.54 (0.46)
1 h plasma glucose, mmol/L	7.38 (2.04)	7.30 (2.03)
2 h plasma glucose, mmol/L	5.81 (1.48)	5.76 (1.46)
HbA_{1c}, mmol/mol	35.3	35.1

† ≥102 cm for men and ≥88 cm for women.

‡ As commonly defined by an FPG≥5.6 mmol/L (100mg/dL) or HbA_{1c} ≥39 mmol/mol (5.7%)

ESM Table 2 – Comparison of those excluded due to missing laboratory with those not excluded.

Characteristic	Exclusions for Missing Glycemic Markers			
	Yes (N=77; 6.2%)		No (N=1174)	
	Mean or %	SD	Mean or %	SD
Age (years)	59.4	10.5	59.1	9.1
Sex (women, %)	74.0		60.9	
Race (white, %)	77.6		78.1	
University education (%)	61.0		66.4	
Obesity (body mass index≥30kg/m^2, %)	26.3		22.7	
Fasting plasma glucose (mmol/L)	5.47	0.53	5.56	0.46
Glycated hemoglobin (mmol/mol)	34.6		35.2	

Characteristic	Exclusions for Missing Insulin			
	Yes (N=121; 10.3%)		No (N=1053)	
	Mean or %	SD	Mean or %	SD
Age (years)	58.8	8.8	59.1	9.1
Sex (women, %)	53.7		61.7	
Race (white, %)	81.7		77.6	
University education (%)	69.4		66.0	
Obesity (body mass index≥30kg/m^2, %)	28.1		22.1	
Fasting plasma glucose (mmol/L)	5.53	0.51	5.57	0.46
Glycated hemoglobin (mmol/mol)	36.0		35.2	

ESM Table 3 – Stages of type 2 diabetes based on fasting plasma glucose (FPG) and 1 h plasma glucose (1 h PG) or FPG and glycated haemoglobin (HbA_{1c})

Stages defined by	Stage 0 (Normal)	Stage 1	Stage 2	Stage 3	Stage 4 (Diabetes)
**FPG/1 h PG**					
Only PG 6.7	FPG<5.6 *and* 1 h PG <6.7	FPG 5.6-6.0 *and* 1 h PG 6.7-8.5	FPG 6.1-6.9 *or* 1 h PG 8.6-11.5	FPG 6.1-6.9 *and* 1 h PG 8.6-11.5	FPG≥7.0 *or* 1 h PG≥11.6
Clinical score with PG	10 year risk† <10% *or* PG as above	10 year risk† ≥10% *and* labs as above	10 yearr risk† ≥10% *and* labs as above	10 year risk† ≥10% *and* labs as above	FPG≥7.0 *or* 1 h PG≥11.6
**FPG/HbA_{1c}**					
Only PG	FPG<5.6 *and* HbA_{1c} <39	FPG 5.6-6.0 *or* HbA_{1c} 39-41	FPG 6.1-6.9 *or* HbA_{1c} 42-47		FPG ≥7.0 *or* HbA_{1c} ≥48
Clinical score with FPG and HbA_{1c}	10 year risk† <10% *or* labs as above	10 yearr risk† ≥10% *and* labs as above	10 year risk† ≥10% *and* labs as above		FPG ≥7.0 *or* HbA_{1c} ≥48

† By a clinical score to detect incident diabetes. FPG and 1 h PG are presented in mmol/L, HbA_{1c} in mmol/mol.

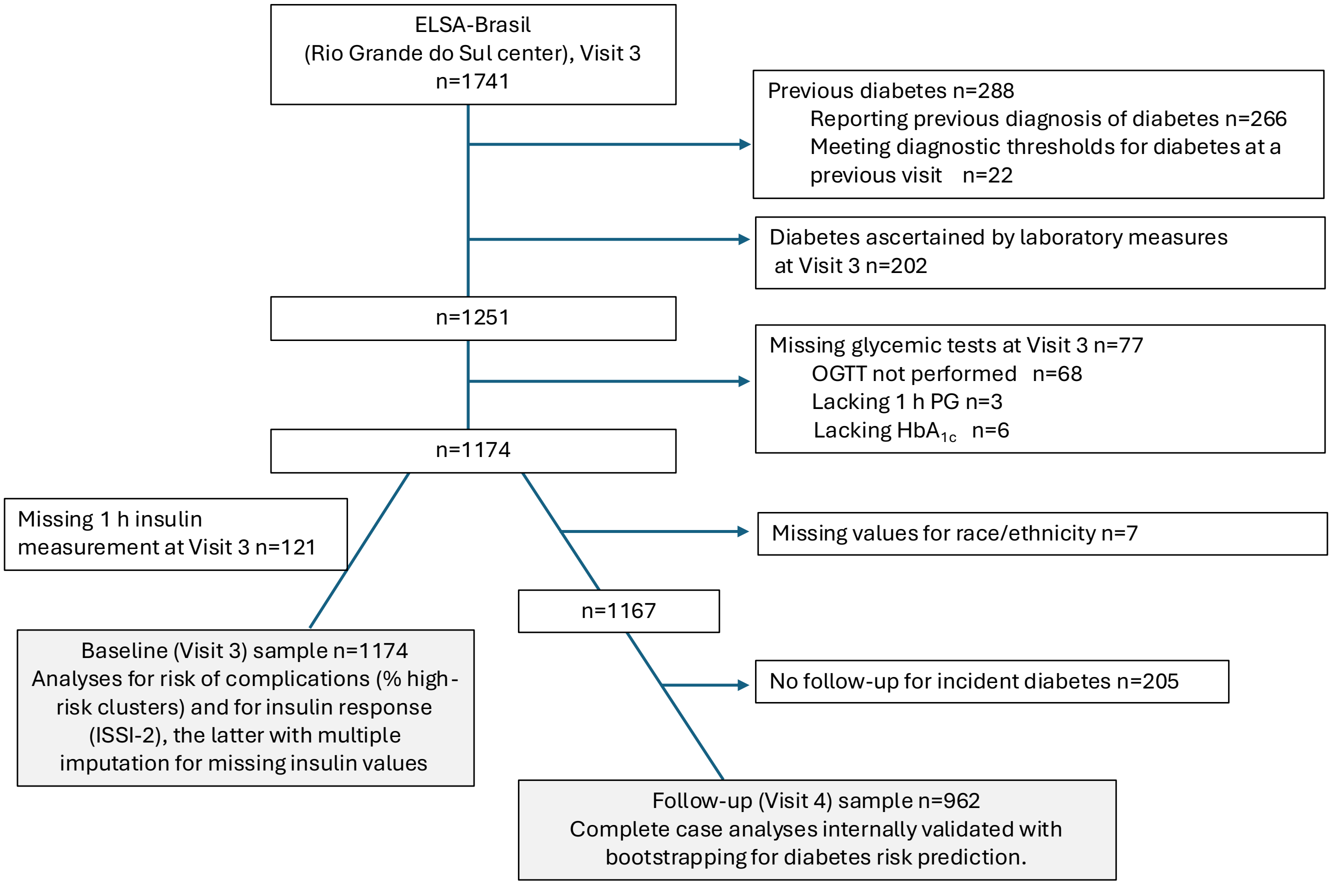

ESM Fig. 1 – Study sample flow diagram.

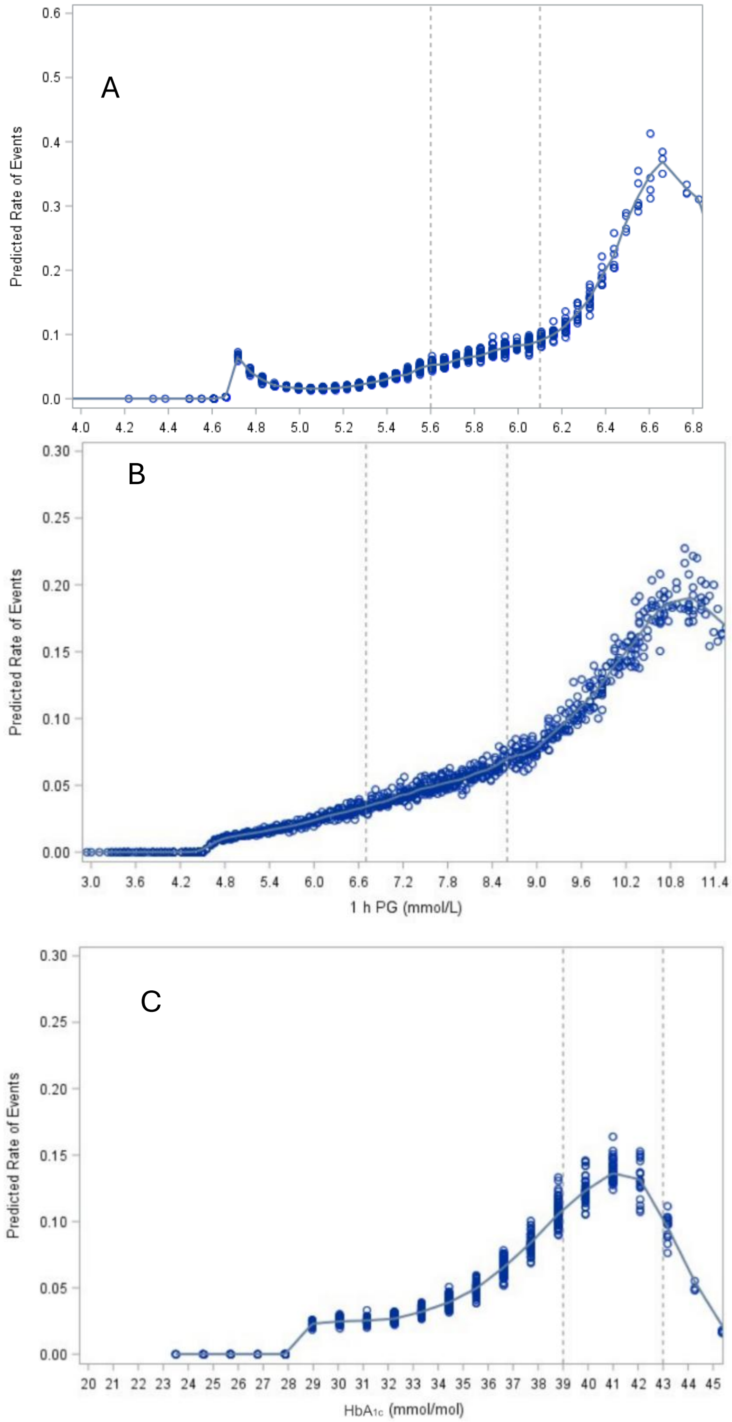

ESM Fig. 2 – Risk of incident diabetes across the spectrum of each glycemic marker used to create stages. A) fasting plasma glucose; B) 1 h plasma glucose; C) HbA_{1c}.

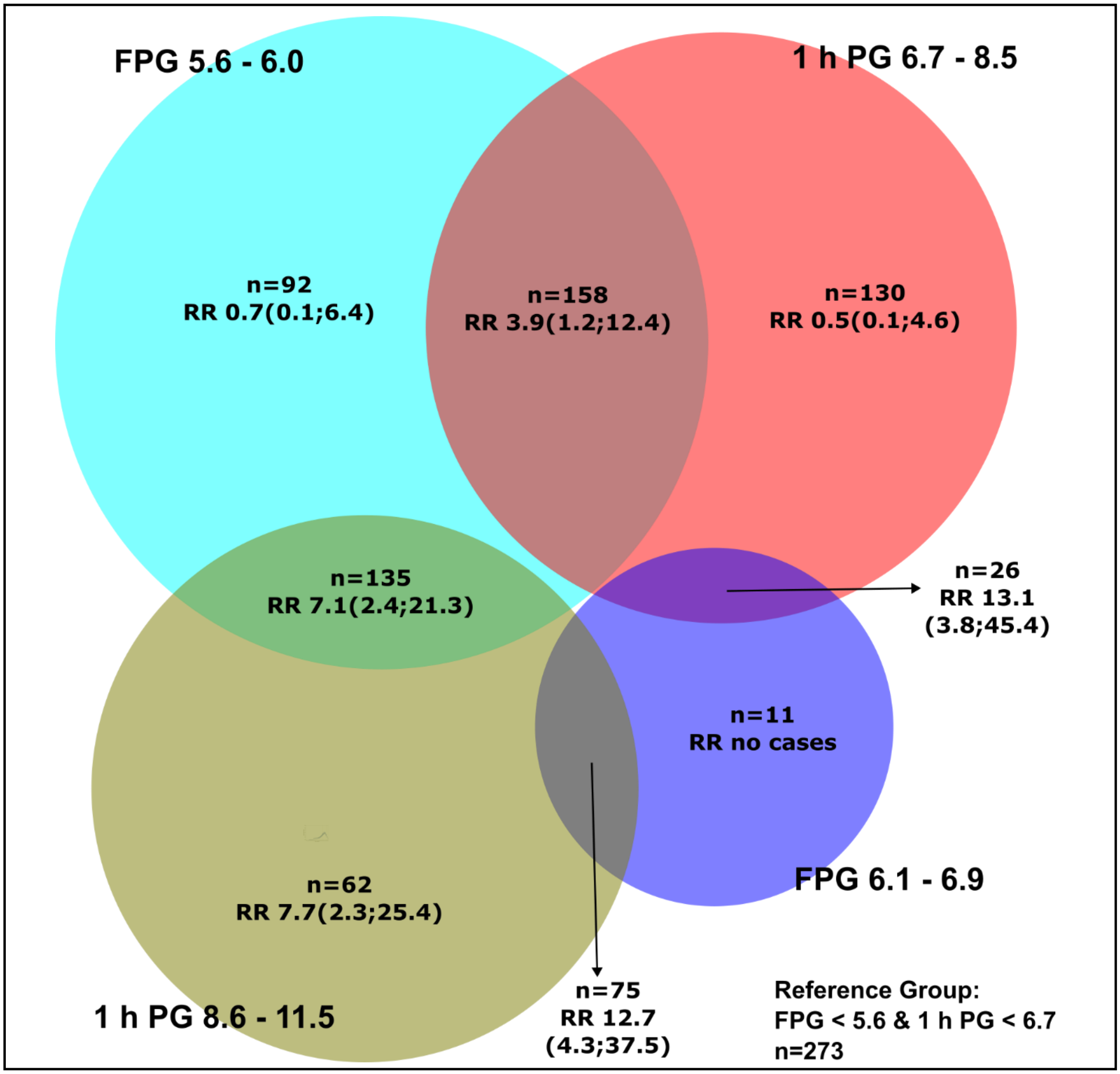

ESM Fig. 3 – Venn diagram to permit consideration of potential stages based on fasting plasma glucose (FPG) and 1 h plasma glucose (1 h PG). The area within the graph represents the total sample, with the white area being participants with low levels of both FPG (<5.6 mmol/L) and 1 h PG (<6.7 mmol/L). The circles represent those with an FPG at mild (5.6-6.0 mmol/L) or moderate (6.1-6.9 mmol/L) and 1 h PG at mild (6.7-8.5 mmol/L) or moderate (8.6-11.5 mmol/L) levels, these being present either in isolation or overlapping. The size of circles is proportional to the percentage of the sample included, with the size of the overlapping and isolated portions being approximate. The number of participants and their relative risk of developing type 2 diabetes over follow-up are shown for each group.

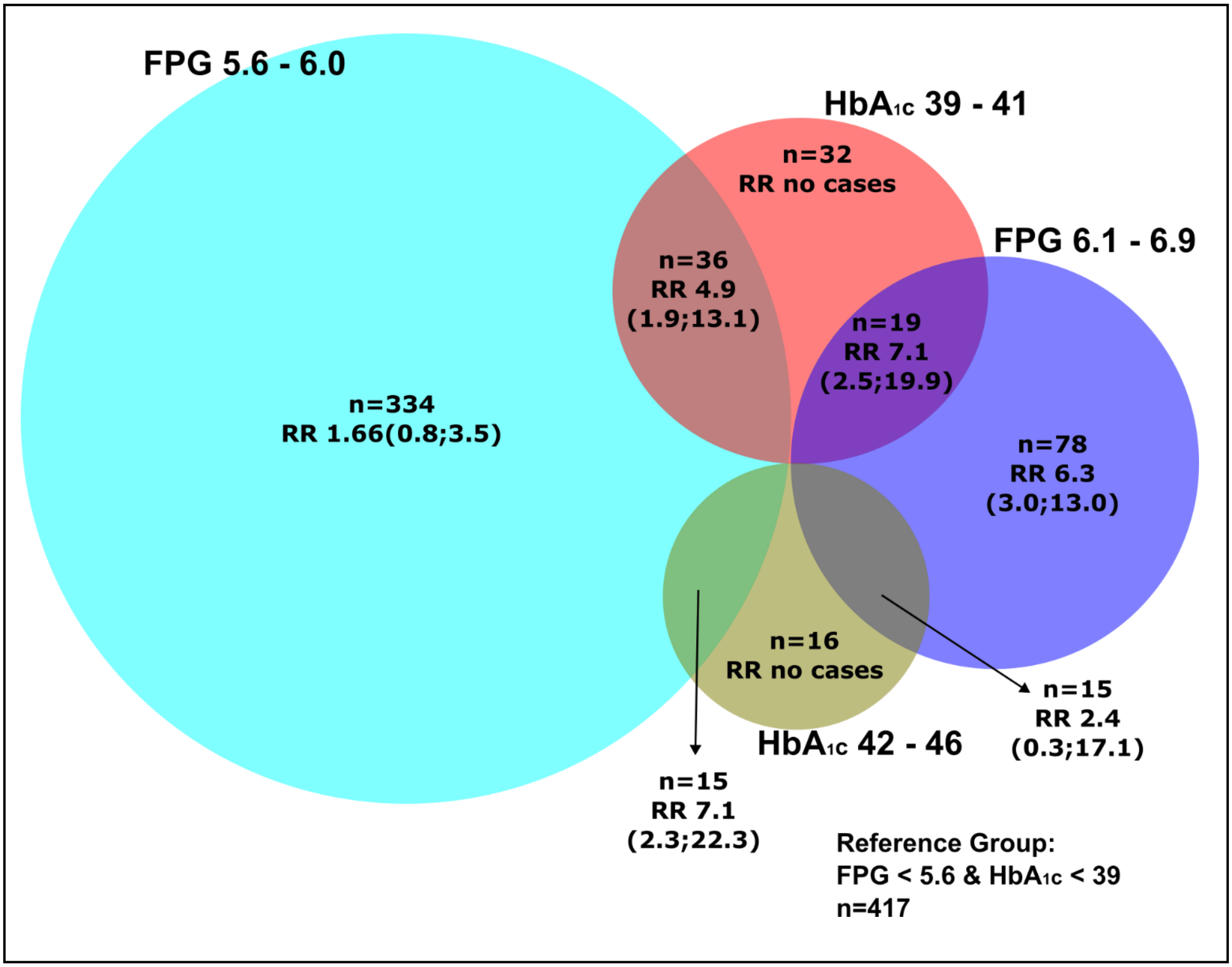

ESM Fig. 4 – Venn diagram to permit consideration of potential stages based on fasting plasma glucose (FPG) and glycated haemoglobin (HbA_{1c}). The area within the graph represents the total sample, with the white area being participants with low levels of both FPG (<5.6 mmol/L) and HbA_{1c} (<39 mmol/mol). The circles represent those with an FPG at mild (5.6-6.0 mmol/L) or moderate (6.1-6.9 mmol/L) and HbA_{1c} at mild (39-41 mmol/mol) or moderate (42-46mmol/mol) levels, these being present either in isolation or overlapping. The size of circles is proportional to the percentage of the sample included, with the size of the overlapping and isolated portions being approximate. The number of participants and their relative risk of developing type 2 diabetes over follow-up are shown for each group.

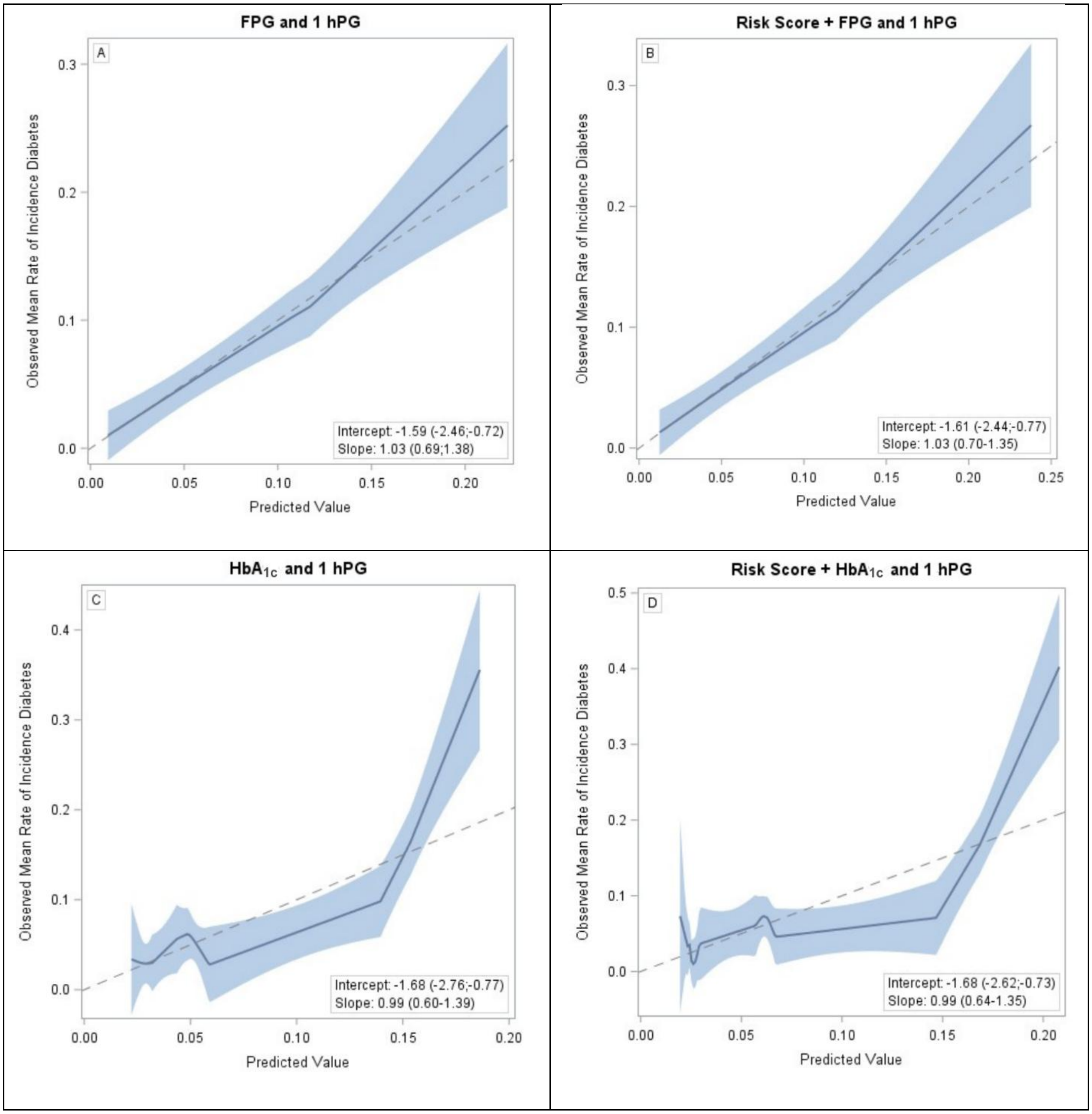

ESM Fig. 5 – Calibration plots for models of stages in the prediction of incident diabetes for the four approaches to staging.
